# The risk of pulmonary tuberculosis after traumatic brain injury

**DOI:** 10.1038/s41598-021-87332-6

**Published:** 2021-04-09

**Authors:** Hsin-Yueh Liu, Kuang-Ming Liao, Fu-Wen Liang, Yi-Chieh Hung, Jhi-Joung Wang, Te-Chun Shen, Chung-Han Ho

**Affiliations:** 1 Department of Internal Medicine, Chi Mei Medical Center, Chiali, Taiwan; 2 Department of Public Health, College of Health Sciences, Kaohsiung Medical University, Kaohsiung, Taiwan; 3 Department of Medical Research, Kaohsiung Medical University Hospital, Kaohsiung, Taiwan; 4 Research Center for Environmental Medicine, Kaohsiung Medical University, Kaohsiung, Taiwan; 5 Department of Neurosurgery, Department of Surgery, Chi-Mei Medical Center, Tainan, Taiwan; 6 Department of Recreation and Healthcare Management, Chia Nan University of Pharmacy and Science, Tainan, Taiwan; 7 Department of Medical Research, Chi Mei Medical Center, No 901, Zhonghua Road, Yongkang District, Tainan City 710, Taiwan; 8 Department of Anesthesiology, Chi Mei Medical Center, Tainan City, Taiwan; 9 AI Biomed Center, Southern Taiwan University of Science and Technology, Tainan City, Taiwan; 10 School of Medicine, China Medical University, Taichung, Taiwan; 11 Division of Pulmonary and Critical Care Medicine, Department of Internal Medicine, China Medical University Hospital, No. 2, Yude Road, Taichung, 404 Taiwan

**Keywords:** Tuberculosis, Brain injuries, Population screening

## Abstract

After traumatic brain injury (TBI), an inflammatory response in the brain might affect the immune system. The risk of pulmonary infection reportedly increases in patients with TBI. We aimed to evaluate the risk of tuberculosis (TB) in patients with TBI in Taiwan. All participants were selected from the intensive care unit (ICU). Patients with TBI were defined as patients in ICU with intracranial injury, and comparison cohort were patients in ICU without TBI diagnosis. There was a significant difference in TB risk between the patients with TBI and the comparison cohort according to age and the Charlson’s comorbidity index (CCI) score. Thus, we divided patients based on CCI into three groups for further analysis: mild (CCI = 0), moderate (CCI = 1/2), severe (CCI > 2). Mild-CCI group had a lower TB incidence rate (0.74%) and longer time to TB development (median: 2.43) than the other two groups. Moderate-CCI group had 1.52-fold increased risk of TB infection (*p* < 0.0001) compared with mild-CCI group. In the severe-CCI group, patients aged ≥ 80 years had 1.91-fold risk of TB compared with mild-CCI group (*p* = 0.0481). Severe-CCI group had significantly higher mortality than the mild-CCI group (*p* = 0.0366). Patients with TBI and more comorbidities had higher risk of TB infection with higher mortality rate.

## Introduction

Tuberculosis (TB) is one of the top ten causes of death in the world and remains the leading cause of death among infectious diseases; a total of 1.4 million people died from TB in 2019^1^. Host factors are important for pulmonary TB infection, and the risk factors include age^2^, use of oral glucocorticoids^3^ or inhalation corticosteroids^4^, diabetes mellitus^5,6^, administration of tumor necrosis factor (TNF-α) inhibitors^7^, organ transplantation^8^, substance abuse^9^, smoking^10^, silicosis^11^, malignancy^12^, chronic renal disease^13^, gastric surgery^14^, liver cirrhosis^15^ and chronic obstructive pulmonary disease^16^. According to WHO's Global Tuberculosis Report, the estimated deaths due to TB were approximately 1.4 million people in 2019^17^. From 2011 to 2014, Taiwan was a country moderately burdened by TB, and the incidence of the disease was about 48–55 per 100,000 people^18^.

Traumatic brain injury (TBI) is a global major cause of mortality and morbidity. After patients could survive from TBI, they may develop systemic immunosuppression and a high risk of bacterial lung infections^19^. Following TBI, the mechanisms of bacterial lung infections include cytokine release, HMGB1 release, and activation of the lymphatic system. The microglia and astrocytes in the brain initiate inflammatory activity and activate the neutrophils to adhere to blood–brain barrier, resulting in cell breakdown and increased permeability. Furthermore, TBI also increases neutrophil and inflammatory cytokines, including TNF-α, interleukin (IL)-1, and IL-6, within the pulmonary tissue^19^.

Following TBI, the inflammatory response might affect the health status. The brain cells secrete cytokines and chemokines, activate the endothelial cells and microglial cells, and result in the migration of systemic neutrophils, lymphocytes, and monocytes into the injured brain^20,21^. Immunosuppression in brain injury is rapid and profound^22^. In the animal model, lymphocyte function was suppressed after brain injury, and the rate of pulmonary infections was significantly higher in the brain injury group compared with the control group^23^. In this study, we hypothesized that the changing immune status after TBI not only increases the risk of lung infections, but also increases the susceptibility of TB infection. Considering the association between level of consciousness and the inflammatory response, patients with TBI and intracranial injury may have a higher inflammatory response than patients without. Therefore, the aim of our study was to assess the risk of TB and the subsequent mortality among patients with TBI and intracranial injury, compared with patients without.

## Methods

### Data source

According to the claims of Taiwan’s national health insurance program, Taiwan established a medical claims database, National Health Insurance Research Database (NHIRD), for research propose. Almost 99% of inpatient and outpatient medical benefit claims for Taiwan’s 23 million residents were covered by NHIRD (http://nhird.nhri.org.tw/en/Data_Subsets.html). The database comprises detailed information regarding clinical visits for each insured person, including date of diagnosis; diagnostic codes according to the International Classification of Diseases, Ninth Revision, Clinical Modification (ICD-9-CM); payments for consultations; and prescription details. This study was conducted in compliance with the Declaration of Helsinki and has been approved by the Research Ethics Committee of Chi Mei Hospital (IRB permit number: 10803-E01). The requirement for informed consent was also waived by the approval of the Research Ethics Committee of Chi Mei Hospital.

### Patient selection and definition

**Figure 1:**
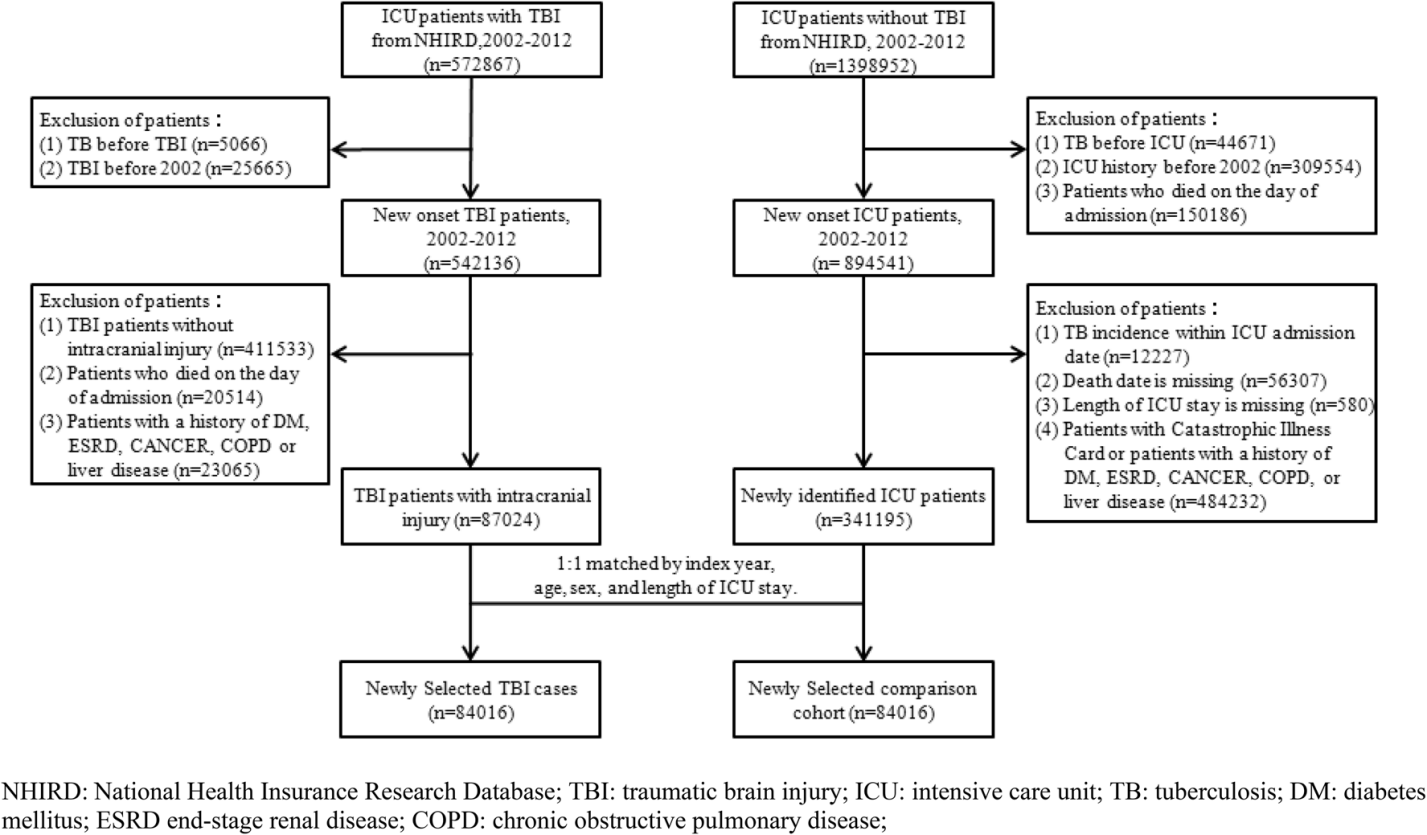
The flowchart for patients’ selection.

All study participants were selected from the admission records of patients admitted in the intensive care unit (ICU) between 2002 and 2012. Patients with TBI were defined as inpatients with a diagnosis code of TBI (ICD-9-CM codes: 800.0–801.9, 803.0–804.9, 850.0–854.1, and 959.01). The patients with TBI were either classified as patients without intracranial injury (ICD-9-CM: 800.0, 800.5, 801.0, 801.5, 803.0, 803.5, 804.0, 804.5, 850.0, 850.1, 850.5, 850.9, 854.0) or patients with intracranial injury (ICD-9-CM: 800.1-800.4, 800.6-800.9, 801.1-801.4, 801.6-801.9, 803.1-803.4, 803.6-803.9, 804.1-804.4, 804.6-804.9, 850.2-850.4, 850.6-850.8, 851-853, 854.1, 959.01), only those with intracranial injury were selected in this study. To understand the potential for TB development in patients with TBI with a more severe inflammatory response, those with intracranial injury were selected in this study. To ensure inclusion of new onset TBI, patients with a history of TBI diagnosis before 2002 were excluded. The comparison cohort were identified as patients without a TBI diagnosis but with ICU admission records in NHIRD. Considering the potential confounding bias that affects TB incidence, patients with a history of diabetes mellitus (DM), end-stage renal disease (ESRD), chronic obstructive pulmonary disease (COPD), cancer, or liver diseases were excluded from the study. To estimate the risk for new-onset TB, all patients with a history of TB before the date of hospital admission or diagnosis of TBI were excluded from both the case cohort and the comparison cohort. Additionally, patients who died on the day of admission were also excluded. For reducing potential selection bias, the comparison cohort was assembled by matching a patient with TBI and one without on index year, age, sex, and length of ICU stay. Finally, a total of 168,032 study participants (84,016 patients with TBI and 84,016 in the comparison cohort) were included in our study. Furthermore, to estimate the association between risk of TB and potential severity among patients with TBI, the Charlson’s comorbidity index (CCI) score was used to further classify the participants into three groups: mild (CCI score = 0), moderate (CCI score = 1 or 2), and severe (CCI score > 2). The CCI score is commonly used as a surrogate to patients’ disease severity^24,25^. Comorbidities measured using the CCI score were based on the 1-year medical records prior to the date of TBI diagnosis; the CCI score has been used in several studies of TBI^26,27^. The flowchart for patients’ selection is presented in Fig. 1.

### Measurements

The main event in this study was TB, which was identified using the ICD-9-CM codes, 010-012 and 010-018, with at least a two-time diagnosis (within 6 months) in an outpatient clinic or during hospital admission, according to the records of the NHI reimbursement datasets. Additionally, to reduce potential misclassification bias, patients with prescriptions of anti-TB drugs, including isoniazid, rifampin, pyrazinamide, ethambutol, were used to confirm the TB diagnosis. All study participants were followed-up until new-onset TB infection, death, or the end date of the study, December 31 2013. Demographic variables (age and sex) and clinical characteristics, including the length of hospital stay (LOS) and the length of ICU stay, were used as potential confounders to estimate the TB and mortality risk. The secondary outcome was the mortality in patients with TB. Mortality was defined as having a death record in the inpatient claim dataset; or outpatient claim dataset; or withdrawal from the insurance program and without reenrollment within 180 days of the last health care visit.

### Statistical analysis

Frequency with percentage and median with interquartile range (IQR) are used to present categorical variables and continuous variables, respectively. The distribution differences in age, sex, mortality, and TB between the study cohorts were examined using the Pearson’s chi-squared test. For continuous variables, including LOS, length of ICU stay, time to TB development, and time to death, Wilcoxon rank-sum test was used to compare the distribution difference between the study groups. Additionally, the cumulative incidence rate of TB was presented using the Kaplan–Meier failure plot, with the log-rank test to compare the trend difference.

Cox proportional-hazards regression model was used to estimate the relative risk of TB between patients with TBI and those without after adjusting for age, sex, LOS, the length of ICU stay, and level of CCI for overall model and the stratified model which excluding the stratified variable (Table 2). Subgroups analyses form Cox regressions were also performed by stratifying according to the CCI modalities (mild, moderate, severe; Table 4). In addition, the mortality risk of moderate- and severe- CCI compared with mild-CCI among TBI patients with TB were also estimated using Cox regression after adjusting for age, sex, LOS, and the length of ICU stay for overall and the age- and sex- stratified model which excluding the stratified variable (Table 5). The Schoenfeld residuals approach was used to check the proportional hazards assumption. SAS 9.4 (SAS Institute, Inc, Cary, NC, USA) was used to perform all statistical analyses. Kaplan–Meier curves were generated using Stata version 12 (Stata Corp, College Station, TX, USA). All significance levels were set at a *p* value < 0.05.

## Results

The baseline information for the TBI and non-TBI group is presented in Table 1. There were significant differences between the two groups regarding incidence of TB, time to TB diagnosis, death, time to death, and CCI (*p* < 0.05). Additionally, the incidence risk of TB was not significantly different between the TBI and non-TBI groups (Table 2); however, TB risk was significantly associated with increasing age among all study participants. Further, the stratified analysis indicated that in patients aged ≥ 80 years, the TBI group had a lower risk of TB compared with the non-TBI group (hazard ratio (HR): 0.63; 95% confidence interval (CI): 0.49–0.81; *p* = 0.0003). Additionally, patients with TBI and mild-CCI had a lower risk of TB than patients without TBI (HR: 0.78; 95% CI 0.68–0.88; *p* = 0.0001). However, patients with TBI and moderate-CCI had 1.25-fold risk of TB incidence (95% CI 1.01–1.56; *p* = 0.0397), compared with patients without TBI. The mortality risk between patients with TBI and non-TBI group did not show the statistical significant for overall and the subgroup of genders. Patients with TBI for patients aged ≥ 50 years, moderate-CCI, and severe-CCI had significant higher mortality risk than patients without TBI. However, compared with non-TBI patients, TBI patients show lower risk of mortality in patients aged < 50 years and mild-CCI group (Table 2).

**Table 1:** The baseline information of patients without TBI and with intracranial injury in ICU.

	ICU patients without TBI N = 84,016	TBI patients with intracranial injury in ICU N = 84,016	*p* value
**Age at entry, years**			
< 20	13,942 (16.59)	13,942 (16.59)	0.9145
20–35	17,942 (21.36)	18,108 (21.55)	
35–50	16,293 (19.39)	16,281 (19.38)	
50–65	16,018 (19.07)	16,006 (19.05)	
65–80	13,798 (16.42)	13,658 (16.26)	
≧80	6023 (7.17)	6021 (7.17)	
**Sex**			
Male	56,416 (67.15)	56,416 (67.15)	1.0000
Female	27,600 (32.85)	27,600 (32.85)	
LOS, median(IQR)	11 (6–20)	11 (7–20)	0.0004
Length of ICU, median(IQR)	3 (2–6)	3 (2–6)	0.6545
**Outcome**			
TB	793 (0.94)	702 (0.84)	0.0181
Time to TB, median (IQR)	1.94 (0.54–4.13)	2.30 (0.94–4.65)	0.0413
Death	11,710 (13.94)	10,242 (12.19)	< .0001
Time to Death, median(IQR)	2.23 (0.62–4.68)	1.87 (0.55–4.10)	< .0001
**Level of CCI**			
Mild-CCI, CCI = 0	53,741 (63.97)	74,842 (89.08)	< .0001
Moderate-CCI, CCI = 1 or 2	25,589 (30.46)	7142 (8.50)	
Severe-CCI, CCI > = 3	4686 (5.58)	2032 (2.42)	

*CCI* Charlson’s comorbidity index, *ICU* intensive care unit, *LOS* length of stay, *SD* standard deviation, *TBI* traumatic brain injury.

**Table 2:** The risk of tuberculosis and mortality in patients with intracranial injury compared to patients without TBI in ICU for whole model and the stratified models.

	TB incidence risk				Mortality risk			
	Adjusted HR^a^ (95% C.I.)	*p* value	Adjusted HR^b^ (95% C.I.) of the stratified variable	*p* value	Adjusted HR^a^ (95% C.I.)	*p* value	Adjusted HR^b^ (95% C.I.) of the stratified variable	*p* value
**Overall**								
TBI versus Non-TBI	0.90 (0.80–1.00)	0.0512	0.90 (0.80–1.00)	0.0512	0.98 (0.95–1.01)	0.1854	0.98 (0.95–1.01)	0.1854
**Age at entry, years**								
< 20	0.43 (0.32–0.60)	< 0.0001	1.30 (0.74–2.27)	0.3669	0.36 (0.33–0.39)	< 0.0001	0.56 (0.48–0.65)	< 0.0001
20–35	1.00 (Ref.)		1.02 (0.74–1.40)	0.9175	1.00 (Ref.)		0.49 (0.45–0.53)	< 0.0001
35–50	1.60 (1.30–1.97)	< 0.0001	1.09 (0.82–1.45)	0.5641	1.22 (1.16–1.29)	< 0.0001	0.86 (0.79–0.93)	< 0.0001
50–65	2.34 (1.92–2.86)	< 0.0001	0.97 (0.74–1.26)	0.7949	1.45 (1.37–1.52)	< 0.0001	1.15 (1.06–1.24)	0.0006
65–80	5.66 (4.71–6.80)	< 0.0001	0.85 (0.70–1.03)	0.0887	3.41 (3.26–3.58)	< 0.0001	1.32 (1.25–1.39)	< 0.0001
≧80	10.31 (8.43–12.61)	< 0.0001	0.63 (0.49–0.81)	0.0003	8.67 (8.26–9.10)	< 0.0001	1.09 (1.03–1.16)	0.0026
**Sex**								
Male	2.55 (2.23–2.91)	< 0.0001	0.91 (0.81–1.03)	0.1295	1.39 (1.35–1.43)	< 0.0001	0.98 (0.94–1.01)	0.1805
Female	1.00 (Ref.)		0.84 (0.65–1.08)	0.1703	1.00 (Ref.)		0.98 (0.93–1.04)	0.4964
**Level of CCI**								
Mild-CCI, CCI = 0	1.00 (Ref.)		0.78 (0.68–0.88)	0.0001	1.00 (Ref.)		0.79 (0.76–0.82)	< 0.0001
Moderate-CCI, CCI = 1 or 2	1.02 (0.90–1.15)	0.7974	1.25 (1.01–1.56)	0.0397	1.39 (1.35–1.44)	< 0.0001	1.43 (1.36–1.50)	< 0.0001
Severe-CCI, CCI > = 3	1.06 (0.86–1.32)	0.5792	0.93 (0.60–1.43)	0.7260	1.42 (1.34–1.49)	< 0.0001	1.29 (1.17–1.43)	< 0.0001

*TBI* traumatic brain injury, *ICU* intensive care unit, *CCI* Charlson’s comorbidity index, *HR* hazard ratio, *CI* confidence interval.

^a^Adjusted by the variables of age, gender, the length of stay, the length of ICU stays, and level of CCI.

^b^Adjusted by the variables of age, gender, the length of stay, the length of ICU stays, and level of CCI, but excluding the stratified variable in the model.

**Figure 2:**
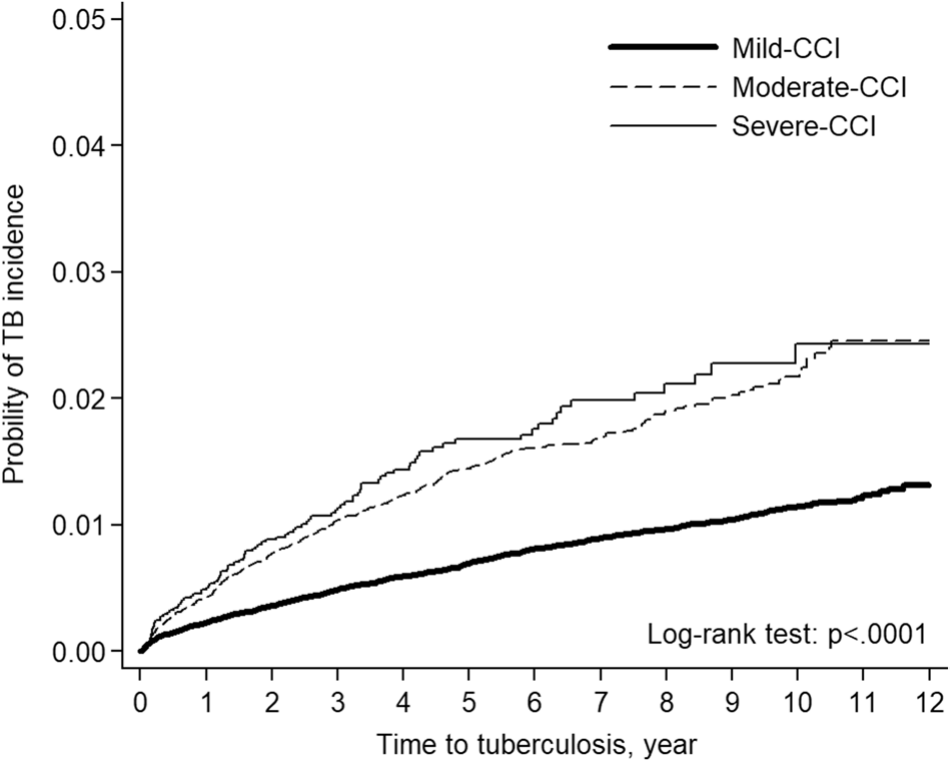
The cumulative incidence rate of TB among the patients with TBI with different levels of CCI.

To understand the baseline difference among patients with TBI with different levels of CCI, the baseline information of the patients with TBI and mild-CCI, moderate-CCI, and severe-CCI is shown in Table 3. There was a significant difference between the three CCI groups regarding the age of the patients with TBI (*p* < 0.05). Patients with moderate-CCI and severe-CCI were significantly older than those with mild-CCI. The severe-CCI group had a longer LOS and length of ICU stay, compared with the other two groups. Furthermore, the mild-CCI group had a lower TB incidence rate (0.74%) and longer time to TB (median: 2.43; IQR: 0.97–4.89) than the other two groups. The moderate-CCI and severe-CCI groups had a higher percentage of TB incidence rate (1.69% and 1.48%, respectively; *p* < 0.0001) and higher mortality rate (32.48% and 31.05%, respectively) than the mild-CCI group. Figure 2 illustrates the difference in the cumulative incidence rate of TB among the patients with TBI with different levels of CCI.

**Table 3:** The baseline information among TBI patients with different Charlson’s Comorbidity Index groups.

Variables	Mild-CCI (N = 74,842)	Moderate-CCI (N = 7142)	Severe-CCI (N = 2032)	*p* value
Age at entry, mean ± SD	43.21 ± 22.26	60.70 ± 22.17	58.00 ± 21.82	< 0.0001
**Age at entry, n** (**%)**				
< 20	13,350 (17.84)	449 (6.29)	143 (7.04)	< 0.0001
20–34	17,208 (22.99)	687 (9.62)	213 (10.48)	
35–49	15,057 (20.12)	920 (12.88)	304 (14.96)	
50–64	14,196 (18.97)	1356 (18.99)	454 (22.34)	
65–79	10,807 (14.44)	2259 (31.63)	592 (29.13)	
≧80	4224 (5.64)	1471 (20.60)	326 (16.04)	
**Sex, n (%)**				
Male	50,308 (67.22)	4743 (66.41)	1365 (67.18)	0.3800
Female	24,534 (32.78)	2399 (33.59)	667 (32.82)	< 0.0001
LOS, median (IQR)	11 (7–18)	19 (9–38)	29 (15–48)	< 0.0001
Length of ICU, median (IQR)	3 (2–6)	5 (2–16)	7 (3–15)	< 0.0001
**Outcome**				
TB, n (%)	551 (0.74)	121 (1.69)	30 (1.48)	< 0.0001
Time to TB, median (IQR)	2.43 (0.97–4.89)	1.88 (0.93–3.93)	1.81 (0.93–3.30)	0.0669
Death, n (%)	7291 (9.74)	2320 (32.48)	631 (31.05)	< 0.0001
Time to death, median (IQR)	1.97 (0.53–4.31)	1.62 (0.56–3.48)	1.93 (0.76–3.95)	< 0.0001

*CCI* Charlson’s comorbidity index, *ICU* intensive care unit, *LOS* length of stay, *TB* tuberculosis, *TBI* traumatic brain injury, *IQR* interquartile range, *SD* standard deviation.

Table 4 shows the overall and stratified TB incidence rate per 10,000 person-year, and HRs of TB among the patients with TBI in different CCI groups. The incidence of TB in patients with TBI and mild-CCI, moderate-CCI, and severe-CCI were 73.62, 169.42, and 147.64 per 10,000 person-year, respectively. Patients with TBI and moderate- and severe-CCI had a higher incidence risk of TB compared with the mild-CCI group. Patients with TBI and moderate-CCI had a 1.52-fold risk of TB (95% CI 1.24–1.87; *p* < 0.0001), compared with those with mild-CCI. For patients with TBI and severe-CCI, the adjusted HR was 1.24-fold (95% CI 0.85–1.80; *p* = 0.2680) than those in the mild-CCI group. For different age groups, patients with TBI in the moderate-CCI group had a higher TB incidence; however, patients in the severe-CCI group aged ≥ 80 years had the highest TB incidence rate (337.42 per 10,000 person-year). Compared with the mild-CCI group, patients in the moderate-CCI group aged 50–64 years and 65–79 years had a significant relative risk of 1.94 (95% CI 1.23–3.05; *p* = 0.0041) and 1.65 (95% CI 1.20–1.26; *p* = 0.0018), respectively. In the severe-CCI group, patients with TBI aged ≥ 80 years showed significant TB risk compared with those in the mild-CCI group (HR: 1.91; 95% CI 1.01–3.64; *p* = 0.0481).

**Table 4:** Tuberculosis incidence and relative risk among TBI patients with different Charlson’s Comorbidity Index groups.

	Mild-CCI		Moderate-CCI		Severe-CCI		Moderate versus mild		Severe versus mild	
	No. of TB	Incidence rate of TB per 10,000	No. of TB	Incidence rate of TB per 10,000	No. of TB	Incidence rate of TB per 10,000	HR (95% CI)^a^	*p* value	HR (95% CI)^a^	*p* value
Overall	551	73.62	121	169.42	30	147.64	1.52 (1.24–1.87)	< 0.0001	1.24 (0.85–1.80)	0.2680
**Age at entry**										
< 20	25	18.73	3	66.82	0	0.00	1.92 (0.50–7.28)	0.3396	–	
20–34	71	41.26	5	72.78	1	46.95	0.96 (0.37–2.49)	0.9388	0.65 (0.09–4.72)	0.6659
35–49	104	69.07	11	119.57	2	65.79	1.45 (0.76–2.78)	0.2636	0.75 (0.18–3.09)	0.6919
50–64	113	79.60	24	176.99	4	88.11	1.94 (1.23–3.05)	0.0041	0.86 (0.32–2.36)	0.7731
65–79	162	149.90	54	239.04	12	202.70	1.65 (1.20–2.26)	0.0018	1.32 (0.73–2.39)	0.3538
≥ 80	76	179.92	24	163.15	11	337.42	1.06 (0.67–1.69)	0.7983	1.91 (1.01–3.64)	0.0481
**Sex**										
Male	462	91.83	95	200.30	23	168.50	1.45 (1.15–1.83)	0.0015	1.16 (0.76–1.78)	0.4829
Female	89	36.28	26	108.38	7	104.95	1.83 (1.15–2.90)	0.0102	1.53 (0.70–3.36)	0.2873

*CCI* Charlson’s comorbidity index, *TB* tuberculosis, *HR* hazard ratio, *CI* confidence interval.

^a^Adjusted by the variables of age, gender, the length of stay, and the length of ICU stays, but excluding the stratified variable in the model.

Additionally, males presented a higher TB incidence rate than females among the three groups. However, females had a significant relative risk for TB in the moderate-CCI group (HR: 1.83; 95% CI 1.15–2.90; *p* = 0.0102), compared with those in the mild-CCI group. Males also showed a significant relative risk for TB between the moderate-CCI and mild-CCI groups (HR: 1.45; 95% CI 1.15–1.83; *p* = 0.0015).

Furthermore, the relative mortality risk among patients with TBI and TB is shown in Table 5. Patients with TBI in the severe-CCI group showed a significantly higher mortality risk of 1.72 (95%: 1.03–2.86; *p* = 0.0366) than those in the mild-CCI group. Patients aged < 50 years were merged due to less events; however, among those aged 50–64 years, patients in the moderate-CCI group presented a 2.6-fold higher mortality risk than those in the mild-CCI group. Further, patients aged 65–79 years in the severe-CCI group had a significant mortality risk (HR: 2.18; 95% CI 1.02–4.65; *p* = 0.0435), compared with those in the mild-CCI group.

**Table 5:** The mortality risk among patients with traumatic brain injury and tuberculosis.

	Moderate-CCI versus Mild-CCI		Severe-CCI versus Mild-CCI	
	HR (95% CI)^a^	*p* value	HR (95% CI)^a^	*p* value
Overall	1.37 (0.98–1.91)	0.0676	1.72 (1.03–2.86)	0.0366
**Age at entry**				
< 50	0.52 (0.15–1.80)	0.2991	1.48 (0.20–10.85)	0.7022
50–64	2.60 (1.18–5.74)	0.0182	1.03 (0.13–8.32)	0.9773
65–79	1.56 (0.94–2.59)	0.0874	2.18 (1.02–4.65)	0.0435
≧80	0.96 (0.49–1.87)	0.9086	1.92 (0.83–4.42)	0.1268
**Sex**				
Male	1.38 (0.96–1.99)	0.0823	1.74 (0.98–3.09)	0.0567
Female	1.10 (0.84–2.76)	0.8423	1.94 (0.64–5.85)	0.2421

*CCI* Charlson’s comorbidity index, *HR* hazard ratio, *CI* confidence interval, *NHIRD* National Health Insurance Research Database, *TBI* traumatic brain injury, *ICU* intensive care unit, *TB* tuberculosis, *DM* diabetes mellitus, *ESRD* end-stage renal disease, *COPD* chronic obstructive pulmonary disease.

^a^Adjusted by the variables of age, gender, the length of stay, and the length of ICU stays, but excluding the stratified variable in the model.

## Discussion

In our study, we observed that the risk of TB was not significantly different between the patients with TBI and patients without TBI admitted in the ICU. This implies that patients with TBI had similar TB risk compared with the high-risk patients in the ICU. However, we observed that the level of CCI scores might majorly affect the risk of TB development in patients with TBI and those with mild- and moderate-CCI. Furthermore, patients with TBI and moderate- and severe-CCI had a higher TB incidence rate and higher mortality than those with mild-CCI.

The risk of TB increased with age and was higher among males. We observed that patients with TBI aged 50–64 years in the moderate-CCI group had the highest risk of developing TB, compared with those in the mild-CCI group; the risk of mortality was also the highest in the moderate-CCI group. There were no statistically significant differences in mortality among the patients aged ≥ 80 years in the moderate-CCI versus mild-CCI groups, and severe-CCI versus mild-CCI groups. To our knowledge, this is the first study to delineate the relationship between TB and TBI. The possible mechanism for increased risk of TB in patients with TBI was alterations in immune system function after TBI, along with presence of comorbidities.

TBI leads to brain damage via direct or indirect injury. These indirect (secondary or delayed) mechanisms begin with an acute inflammatory response encompassing destruction of the blood–brain barrier and brain cells, tissue swelling, and edema. Subsequently, cell recruitment, and restoration and stimulation of peripheral blood cells and tissue-resident immune cells ensues. The immune mediators, such as chemotactic factors and interleukins, augment the inflammatory response and result in possibly harmful or helpful sequelae of TBI^28^.

Cytokines such as TNF-α and IL-6 are important mediators that initiate and maintain inflammation after TBI. Brain injuries result in immune system suppression and subsequent infection. Immunosuppression in brain injury is profound and may be associated with infection after TBI. Wolach et al.^22^ observed the occurrence of infections in nine (75%) of 12 patients after TBI. These patients with TBI had immune system impairment, which included deficiencies such has neutrophil superoxide release, immunoglobulins, complement system components, properdin, alternate C pathway, T cells, T helper cells, T suppressor cells, and natural killer cells. These immune deficiencies probably contributed to the high rate of infections. Munno et al.^29^ studied the relationship between persistent vegetative status after TBI and immune system. They observed that patients had profound impairment of phagocytosis and killing of monocytes, and low serum levels of interferon gamma. Wolach et al.^30^ also observed impaired humoral immunity in patients with post-comatose unawareness. Patients also had decreased levels of complement system components, hemolytic activity of the classical complement pathway, immunoglobulins, and neutrophil killing activity; however, neutrophil function of chemotaxis, random migration, and superoxide anion release was normal. All of these factors contribute to increased susceptibility of TB infection in immunosuppressed patients with TBI.

After further analysis of the comorbidities via CCI in the patients with TBI, we observed that compared with mild-CCI, the moderate-CCI group had a higher risk of TB between the ages of 35 and 79, and the severe-CCI group had higher risk of TB in patients aged ≥ 80 years. There were no statistically significant differences in TB incidence among patients ≤ 35 years in the moderate- and severe-CCI group, compared with the mild-CCI group. This might have been due to the small number of patients in that group, which provided inadequate statistical power to demonstrate a difference. After stratification by age, each group (moderate-CCI and severe-CCI) had a limited number of patients, and the sample size was too small to have enough statistical power to detect.

Comorbidities play an important role in the severity and mortality in patients with TB. Factors such as advancing age and co-infection with HIV have been linked to mortality in patients with TB infections^31^. CCI is a standardized method to evaluate comorbidities, which was originally used in 1987 to predict 1-year all-cause mortality due to the 17 underlying conditions^24^; it has been validated and widely used clinically.

In our study, we demonstrated that CCI was associated with mortality in the patients who developed TB after TBI, and we observed that patients with TBI aged ≤ 80 years in the moderate-CCI group had higher risk of mortality of developing TB. CCI has been shown to be an independent predictor of mortality and long-term survival. The patients with moderate-CCI had a higher risk of mortality compared with patients with mild-CCI. The same result was observed in the severe-CCI group compared with the mild-CCI group, though only in those aged 65–79 years, due to the small sample size in other age groups. Whether different comorbidities or age have an impact on TB incidence after TBI needs further investigation, and more studies are required to confirm the impact of comorbidities on mortality.

Beside the brain injury itself, it is increasingly evident that a brain injury also changes the systemic immune response in a way that makes patients with TBI more susceptible to infections in the post-injury period; these infections are associated with morbidity and mortality and can be an added challenge to treat patients. Further research to better understand immunosuppression rather than TBI-induced immunosuppression is more important for the development of effective therapeutic strategies for helping these patients^32^. A study has shown that TBI activates the sympathetic nervous system, and further upregulates the expression of programmed cell death-1 on CD4^+^ and CD8^+^ T cells, which, in turn, impairs their function and contributes to immunosuppression^33^.

There are some limitations to our study. Based on the database, we were unable to analyze the type of TBI during hospitalization; the mortality risk may have been related to the type of TBI. We also lacked data regarding functional outcomes and consciousness level after patient discharge. Besides, we could not rule out any long-term mortality that might have been caused by the sequelae associated with TBI, or indirectly, by other diseases. We also did not have the laboratory and imaging data, including brain computed tomography scan.

Despite these limitations, our study enrolled a large number of patients with TBI with different comorbidities, divided them according CCI scores, and underwent long-term follow-up at various hospitals. Therefore, our findings may have broad generalizability to this population and provide valid estimates of the population characteristics.

## Conclusion

Patients with TBI with more comorbidities increased the risk of TB infection. A careful survey and diagnosis are important for determination of a subsequent treatment strategy. The long-term mortality risk in patients with TB after TBI was significantly higher than the corresponding risk in patients with mild-CCI. Our findings might provide some information for clinicians to improve long-term care strategies in elderly patients with TBI.

## References

[1] World Health Organization. *Tuberculosis*. https://www.who.int/news-room/fact-sheets/detail/tuberculosis (2020).

[2] Hochberg NS, Horsburgh CR (2013). Prevention of tuberculosis in older adults in the United States: Obstacles and opportunities. Clin. Infect. Dis..

[3] Jick SS, Lieberman ES, Rahman MU, Choi HK (2006). Glucocorticoid use, other associated factors, and the risk of tuberculosis. Arthritis Rheum..

[4] Dong YH, Chang CH, Lin Wu FL, Shen LJ, Calverley PM, Löfdahl CG, Lai MS, Mahler DA (2014). Use of inhaled corticosteroids in patients with COPD and the risk of TB and influenza: A systematic review and meta-analysis of randomized controlled trials. Chest.

[5] Jeon CY, Murray MB (2008). Diabetes mellitus increases the risk of active tuberculosis: A systematic review of 13 observational studies. PLoS Med..

[6] Baker MA, Harries AD, Jeon CY, Hart JE, Kapur A, Lönnroth K, Ottmani SE, Goonesekera SD, Murray MB (2011). The impact of diabetes on tuberculosis treatment outcomes: A systematic review. BMC Med..

[7] Keane J, Gershon S, Wise RP, Mirabile-Levens E, Kasznica J, Schwieterman WD, Siegel JN, Braun MM (2001). Tuberculosis associated with infliximab, a tumor necrosis factor alpha-neutralizing agent. N. Engl. J. Med..

[8] Akan H, Arslan O, Akan OA (2006). Tuberculosis in stem cell transplant patients. J. Hosp. Infect..

[9] Oeltmann JE, Kammerer JS, Pevzner ES, Moonan PK (2009). Tuberculosis and substance abuse in the United States, 1997–2006. Arch. Intern. Med..

[10] Bates MN, Khalakdina A, Pai M, Chang L, Lessa F, Smith KR (2007). Risk of tuberculosis from exposure to tobacco smoke: A systematic review and meta-analysis. Arch. Intern. Med..

[11] Cowie RL (1994). The epidemiology of tuberculosis in gold miners with silicosis. Am. J. Respir. Crit. Care Med..

[12] Kamboj M, Sepkowitz KA (2006). The risk of tuberculosis in patients with cancer. Clin. Infect. Dis..

[13] Pien FD, Younoszai BG, Pien BC (2001). Mycobacterial infections in patients with chronic renal disease. Infect. Dis. Clin. N. Am..

[14] Bruce RM, Wise L (1977). Tuberculosis after jejunoileal bypass for obesity. Ann. Intern. Med..

[15] Thulstrup AM, Mølle I, Svendsen N, Sørensen HT (2000). Incidence and prognosis of tuberculosis in patients with cirrhosis of the liver. A Danish nationwide population based study. Epidemiol. Infect..

[16] Lee CH, Lee MC, Shu CC, Lim CS, Wang JY, Lee LN, Chao KM (2013). Risk factors for pulmonary tuberculosis in patients with chronic obstructive airway disease in Taiwan: A nationwide cohort study. BMC Infect. Dis..

[17] World Health Organization. *Global Tuberculosis Report*. https://www.who.int/teams/global-tuberculosis-programme/tb-reports/global-tuberculosis-report-2020 (2020).

[18] Taiwan Centers for Disease Control, Department of Health. *Taiwan Tuberculosis Control Report*. https://www.cdc.gov.tw/english/infectionreport.aspx?treeid=3847719104be0678&nowtreeid=ffb51203f16bfe57 (2020).

[19] Hu PJ, Pittet JF, Kerby JD, Bosarge PL, Wagener BM (2017). Acute brain trauma, lung injury, and pneumonia: More than just altered mental status and decreased airway protection. Am. J. Physiol. Lung Cell Mol. Physiol..

[20] Zanier ER, Fumagalli S, Perego C, Pischiutta F, De Simoni M-G (2015). Shape descriptors of the “never resting” microglia in three different acute brain injury models in mice. Intensive Care Med. Exp..

[21] Loane DJ, Kumar A, Stoica BA, Cabatbat R, Faden AI (2014). Progressive neurodegeneration after experimental brain trauma: Association with chronic microglial activation. J. Neuropathol. Exp. Neurol..

[22] Wolach B, Sazbon L, Gavrieli R, Broda A, Schlesinger M (2001). Early immunological defects in comatose patients after acute brain injury. J. Neurosurg..

[23] Miao Y, Zhang M, Nie Y, Zhao W, Huang B, Jiang Z, Shaoxiong Yu, Huang Z, Hongjin Fu (2007). Changes in T lymphocyte subsets after severe traumatic brain injury. Neural Regen. Res..

[24] Charlson ME, Pompei P, Ales KL, MacKenzie CR (1987). A new method of classifying prognostic comorbidity in longitudinal studies: Development and validation. J. Clin. Epidemiol..

[25] Charlson M, Szatrowski TP, Peterson J, Gold J (1994). Validation of a combined comorbidity index. J. Clin. Epidemiol..

[26] Thompson HJ, Dikmen S, Temkin N (2012). Prevalence of comorbidity and its association with traumatic brain injury and outcomes in older adults. Res. Gerontol. Nurs..

[27] Ho CH, Liang FW, Wang JJ, Chio CC, Kuo JR (2018). Impact of grouping complications on mortality in traumatic brain injury: A nationwide population-based study. PLoS ONE.

[28] Lenzlinger PM, Morganti-Kossmann MC, Laurer HL, McIntosh TK (2001). The duality of the inflammatory response to traumatic brain injury. Mol. Neurobiol..

[29] Munno I, Damiani S, Lacedra G, Mastropasqua V, Megna GF (1996). Impairment of non-specific immunity in patients in persistent vegetative state. Immunopharmacol. Immunotoxicol..

[30] Wolach B, Sazbon L, Gavrieli R, Ben-Tovim T, Zagreba F, Schlesinger M (1993). Some aspects of the humoral and neutrophil functions in post-comatose nawareness patients. Brain Inj..

[31] Heunis JC, Kigozi NG, Chikobvu P (2017). Risk factors for mortality in TB patients: A 10-year electronic record review in a South African province. BMC Public Health.

[32] Needham EJ, Helmy A, Zanier ER, Jones JL, Coles AJ, Menon DK (2019). The immunological response to traumatic brain injury. J. Neuroimmunol..

[33] Yang Y, Ye Y, Chen C, Kong C, Su X, Zhang X, Bai W, He X (2019). Acute traumatic brain injury induces CD4^+^ and CD8^+^ T cell functional impairment by upregulating the expression of PD-1 via the activated sympathetic nervous system. NeuroImmunoModulation.

